# High-resolution subtyping of fibroblasts in gastric cancer reveals diversity among fibroblast subsets and an association between the MFAP5-fibroblast subset and immunotherapy

**DOI:** 10.3389/fimmu.2024.1446613

**Published:** 2024-10-25

**Authors:** Hong Wang, Linjun Yang, Wei Chen, Kainan Li, Meng Xu, Xiaobo Peng, Jie Li, Feng Zhao, Bin Wang

**Affiliations:** ^1^ Department of Gastrointestinal Surgery, Shandong Provincial Third Hospital, Cheeloo College of Medicine, Shandong University, Jinan, Shandong, China; ^2^ Department of Oncology, Changhai Hospital, Naval Medical University, Shanghai, China; ^3^ Department of Radiology, Changhai Hospital, Naval Medical University, Shanghai, China; ^4^ Department of Oncology, The Second Affiliated Hospital of Shandong University of Traditional Chinese Medicine, Jinan, Shandong, China; ^5^ Outpatient Department, Changhai Hospital, Naval Medical University, Shanghai, China

**Keywords:** gastric cancer, fibroblast, single cell RNA sequencing, MFAP5, immunotherapy, precision medicine

## Abstract

**Backgrounds:**

Gastric cancer (GC) remains a global health threat due to frequent treatment failures caused by primary or acquired resistance. Although cancer-associated fibroblasts (CAFs) have been implicated in this process, it is still unclear which specific subtype(s) of CAFs hinder T-cell infiltration and promote resistance to immunotherapy.

**Methods:**

We analyzed the GC fibroblast atlas in detail by combining 63,955 single cells from 14 scRNA-seq datasets. We also performed RNA-seq data in a local GC cohort and examined 13 bulk RNA-seq datasets to understand the biological and clinical roles of different CAF subsets. Additionally, we conducted *in vitro* experiments to study the role of specific proteins in GC development.

**Results:**

We identified a total of 17 fibroblast subsets in gastric cancer, nine of which did not fit into the existing CAFs classification. These subsets exhibited significant heterogeneity in distribution and biological characteristics (metabolism, cell-cell interactions, differentiation state), as well as clinical functions such as prognosis and response to immunotherapy. In particular, cluster 6 stood out for its high expression of MFAP5, CFD, and PI16; it was found to be negatively associated with both overall survival and response to immunotherapy in GC. This association was linked to an immunosuppressive microenvironment characterized by an increase in M2 macrophages but higher levels of T cell dysfunction and exclusion—a feature shared by tumors expressing MFAP5. Furthermore, the addition of human recombinant MFAP5 promoted proliferation and migration of HGC-27 cells by inducing the MFAP5/NOTCH1/HEY1 signaling pathway.

**Conclusion:**

We introduce a high-resolution GC fibroblast atlas. The 17 identified fibroblast clusters provide valuable opportunities for gaining deeper biological insights into the relationship between fibroblasts and GC development. Particularly, cluster 6 and its specific marker MFAP5 could serve as prognostic factors in GC and form a foundation for personalized therapeutic combinations to address primary resistance to ICIs.

## Introduction

Gastric cancer (GC) ranks as the fifth most prevalent malignant tumor globally and as the third leading cause of cancer-related mortality in China, which presents a formidable health challenge (1). Although significant advancements have been achieved in unraveling the molecular mechanisms underlying GC and great strides in cancer treatment, the overall survival of GC patients is still poor (2). Chemotherapy is still the standard treatment in GC, though treatment failure frequently occurs due to intrinsic or acquired resistance (3). Over the past decades, immune checkpoint inhibitors have revolutionized the management of lots of types of cancers. However, only a small set of GC patients are sensitive to the ICI and the responders with GC were mainly limited in those with deficient mismatch repair (dMMR)/microsatellite high (MSI-H) (4). Hence, there is a pressing demand for understand the biological mechanism and developing innovative and efficacious cancer treatments to solve the therapeutic resistance.

GC cells growth and metastasis to distant organs rely on the support of the tumor microenvironment (TME), which consists of extracellular matrix (ECM), immune cells, endothelial cells, and fibroblasts (5). These TME components engage in extensive bidirectional communication through cell-to-cell interactions and secreted molecules. Among the components of GC TME, Cancer-associated fibroblasts (CAFs) were one of the most prevalent cell types, primarily originating from resident tissue fibroblasts in response to signals from tumors (6). CAF activation is induced by factors like ECM rigidity, metabolic stress, and signaling molecules such as TGF-β, IL-1, IL-6, and TNF released by both tumor cells and infiltrating immune cells. Upon activation, CAFs increase expression of functional markers, including PDGFRβ and S100A4 and undergo metabolic changes that boost aerobic glycolysis supporting their proliferative and secretory functions. In the meantime, CAFs could also induce anti-tumor drug resistance development by secreting functional proteins and cellular components, such as metalloproteinases and exosomes (7). More importantly, as an important regulator in shaping the TME and tumor-infiltrated immune cells (TIICs), CAFs also contributed to immunotherapy resistance (8).

The inter- and intra-tumoral molecular heterogeneity of GC cancer cells has been extensively studied (9, 10). Rogers et al. discovered that cancer-associated fibroblasts (CAFs) can transfer the Wnt receptor ROR2 to GC cells via cytoneme, promoting tumor survival (11). However, the heterogeneity of CAFs in GC has not been fully explored. Previous studies on breast or pancreatic cancer have identified various subtypes of CAFs, such as vascular CAFs (vCAFs), matrix CAFs (mCAFs), and myofibroblastic CAFs (myCAFs) (12, 13). These subtypes display unique transcriptional profiles, functions, and spatial distribution within the tumor mass. For example, inflammatory CAFs (iCAFs) primarily express inflammatory cytokines such as IL-6, LIF, and CXCL12. This could lead to the recruitment of regulatory T cells (Tregs), increased secretion of TGF-β, and the promotion of an immunosuppressive TME (14). myCAFs are linked to focal adhesion and interactions with the extracellular matrix, showing evidence in enhancing the stemness of tumor cells by secreting senescence-associated secretory phenotype factors IL-6 and IL-8 to resist chemotherapy (15). While antigen-presenting CAFs (apCAFs) are enriched in pathways related to antigen processing and presentation, they can directly interact with and convert naive CD4+ T cells into Tregs in an antigen-specific manner (16). It is important to note that there is evidence revealing further heterogeneity within the existing classification of CAFs, highlighting the need for a more concise classification (17, 18). On the other hand, it is still unclear which specific subtype(s) of CAFs restricts T-cell infiltration into the TME. This restriction could reduce the sensitivity to ICIs, leading to a lack of understanding in how CAFs affect response to ICIs in GC.

While some studies have conducted single-cell RNA sequencing (scRNA-seq) in GC to elucidate specific biological functions of CAF subtypes, discrepancies in their results remain. To address this gap, we integrated major publicly available scRNA-seq datasets to create a comprehensive atlas of GC fibroblasts comprising of 63,955 cells and analyzed their biological diversity.

## Materials and methods

### Integration of scRNA-seq datasets and unsupervised clustering

The design of this study was shown in the schematic diagram in **Supplementary Figure 1**. Initially, we downloaded fourteen 10x single-cell datasets from public databases (**Supplementary Table 1**). For datasets lacking raw count data, we obtained fastq data and processed it to derive count data. Subsequently, these scRNA-seq data was analyzed using Seurat (v4.4.0) in R (v4.1.3). Gene names in the raw count matrix were converted based on information from HGNC (2023-10-01), prioritizing gene names with higher expression levels when duplicates occurred. To create the final expression matrix, only gene names present in at least 10 datasets were retained. Each expression matrix was used to generate a Seurat object for analysis after applying uniform preprocessing criteria: cells with features between 200 and 7000 were retained, while those with mitochondrial gene expression exceeding 20% were removed using a cutoff of 20%. Log normalization was conducted via the NormalizeData method with scale.factors set to 1e4. The top 3000 variable features of the single-cell data were identified using FindVariableFeatures with the vst parameter and their expressions scaled through ScaleData method. Distinct cell clusters were identified by employing FindNeighbors and FindClusters methods at a resolution of 0.5. Dimensionality reduction for visualization purposes was achieved using RunUMAP method. To annotate cell clusters, we utilized the FindAllMarkers method employing Wilcoxon test with logfc.threshold set at 0.25 followed by manual annotation of cell clusters. Default parameters from the Seurat package were used for all methods unless specified otherwise. Fibroblast/CAF clusters were filtered using DCN, ACTA2, and COL1A1 as markers. The selected clusters were merged and batch effects removed with Harmony (v1.2.0). The aggregated single-cell expression matrix of Fibroblast/CAF was analyzed using standard pipelines. PCA coordinates of fibroblast cells were then input into Harmony to correct patient-level batch effects through the RunHarmony method iteratively. After obtaining Harmony embeddings, RunUMAP and FindNeighbors methods with Harmony reductions were applied (19). Final fibroblast/CAF subtypes were identified using the FindClusters method at a resolution of 0.5.

### Gene expression feature and biological pathway enrichment

We conducted enrichment analysis on the differentially expressed genes in each cluster by identifying the top 50 upregulated genes. Using the msigdbr (v7.5.1) package in R, we retrieved KEGG, GO, and HALLMARK gene sets from MSigDB. Subsequently, over-representation analysis (ORA) was performed with the ClusterProfiler (v4.2.2) package to identify enriched pathways for each cluster. Each cluster showed distinct enriched pathways; therefore, we selected the top 2 KEGG pathways for visualization and created a corresponding figure. To assess the metabolic activity at the single-cell level, we utilized scMetabolism (v0.2.1). By employing the FindMarkers function in Seurat, we identified the predominant metabolic pathways within each cluster. The figure below illustrates the two most active metabolic pathways chosen from every cluster. To obtain single-cell transcription factor levels, first download the CollecTF transcription factor database using decoupleR (v2.9.6) and OmnipathR (v3.11.10). Next, infer the activity of transcription factors in single-cell data with viper (v1.28.0). We utilize the FindMarkers method in Seurat to identify highly active transcription factors within each cluster.

### Cell trajectory and differentiation analysis

We validated the trajectory using monocle3 (20), CytoTRACE (21), and Slingshot (22). We obtained the raw count matrix of fibroblast cells and processed it using the Monocle3 pipeline with default parameters following official guidelines. Trajectories were learned using the learn_graph method, and pseudotime was estimated with the order_cells method. Slingshot objects were created based on UMAP embeddings, and pseudotime was determined using slingPseudotime. The single-cell expression matrix was then analyzed with CytoTRACE to predict cell ordering. The unprocessed count matrix of fibroblasts was used for Monocle3 analysis with default parameters. Trajectories were learned and pseudotime estimated through specific methods in each tool. UMAP embeddings were utilized to construct Slingshot objects and infer pseudotime. Additionally, CytoTRACE (v0.3.3) was applied to predict cell ordering based on single-cell data for differentiation trajectories of fibroblast cells. After determining the differentiation status and trajectories with CytoTRACE, we confirmed pseudotime using monocle3 (v 1.3.6) and Slingshot (v 2.2.1). To ensure consistency across tools, CytroRACE values were scaled and negated before analyzing CAF subclusters based on cell infiltration levels relative to expected numbers; higher values indicated greater infiltration.

### Cell contact analysis

We studied interactions of cluster 6 fibroblasts with other cells by selecting datasets GSE249874, Li_2022, Sathe_2020, and Zhao_2023 known for their high abundance of cls_6 fibroblasts. Cell types in each dataset were identified and analyzed using CellChat (v 2.1.2) (23) to explore interactions like Cell-Cell contact, ECM-receptor signaling, and Secreted pathways. The CellChat package utilized the human ligand-receptor interaction database. Initially, we preprocess the expression data using identifyOverExpressedGenes and identifyOverExpressedInteractions methods. Subsequently, we deduce the cell-cell communication network through computeCommunProb and computeCommunProbPathway functions. Finally, we consolidate the cell-cell communication network with the aggregateNet method. Significant interactions had a p-value < 0.01; those present in all datasets were considered promising.

### Analysis the biological and clinical function of identified CAFs cluster in bulk-RNA data

We generated a signature matrix for fibroblasts from scRNA data, similar to the method used in CIBERSORT (24). Using Seurat (4.4.0)’s FindAllMarkers function, we identified markers for all CAF clusters and selected the most significant ones based on p_val_adj < 0.05 and log2FC > 0.5 criteria. From these markers, we created 100 candidate matrices and assessed their stability by calculating the condition number using R’s kappa method; a lower condition number indicates better stability. The matrix with the smallest condition number was chosen as our final signature matrix. Finally, we evaluated CAF cluster infiltration levels in tissue transcriptomes using the CIBERSORT method. All the detail of utilized thirteen public GC bulk RNAsq datasets were presented in **Supplementary Table 2**.

### SCISSOR analysis

We utilized SCISSOR (25) (v 2.0.0) to pinpoint fibroblast cells linked to survival outcomes by analyzing TCGA RNA-seq data and survival details. A grid search was conducted for the alpha parameter, using the default value and a cutoff of 0.20 as suggested by the author. The cutoff of 0.20 indicates that no more than 20% of cells should be selected. Ultimately, we identified 6160 cells correlated with poorer prognosis.

### Patient enrollment and RNA sequencing

This trial was approved by Shanghai Changhai hospital ethics committee and all enrolled patients have provided written informed consent previously by themselves or their legal representatives. We firstly enrolled 95 GC patients who performed surgery in our hospital between 2012 and 2015. For all the archived FFPE tumor tissue samples collected, a tumor cell content greater than 50% was required. Then, RNA was extracted from the tumor samples by using Biozol RNA extraction kit (BW-R7311, Beiwo.co, China) according to the manufacture’s instruction. Then, Qubit RNA Assay kits (Quant-iT™ PicoGreen^®^ dsDNA Assay Kit, Life Technologies) and Agilent 2100 Bioanalyzer were used to measure RNA concentration and RNA integrity number (RIN), respectively. A total of 50 samples were failed to pass the quality control or loss the follow up, and finally 45 samples had sufficient RNA quality to comprise the final local validation cohort. For depletion of ribosomal RNA and library construction, VAHTS^®^ Universal V6 RNA-seq Library Prep Kit for Illumina with Ribo-off rRNA Depletion Kit (Vazyme.co, China) was used. Library concentrations were measured using the Qubit dsDNA HS Assay kit (Life Technologies) and Agilent TapeStation (Agilent). RNA sequencing was performed at the Xuran laboratory, using Illumina NovaSeq 6000 equipment for double-end sequencing. The clinical information of enrolled GC patients in the local cohort was listed in **Supplementary Table 3**.

### Cell culture

HGC-27 cells were purchased from the Cell Bank of the Chinese Academy of Sciences (Shanghai, China) and tested negative for mycoplasma. Cells were grown in an incubator at 5% CO_2_ and 37°C. The cell lines were cultured in DMEM (Gibco, USA) containing L-glutamine, 4.5 g/L D-glucose, and 110 mg/L pyruvate nano, together with 1% penicillinstreptomycin (HYClone, USA) and 10% fetal bovine serum (FBS, Gibco).

### Cell viability assay

Cell viability was assessed using Cell Counting Kit 8 (CCK-8) from Yeasen, China, following the manufacturer’s instructions. HGC-27 cells were seeded in 96-well plates at a density of 5000 cells per well. Then, they were treated with 10 µg/mL human recombinant MFAP5 (hr MFAP5, TargetMol, USA) for 24 hours. Subsequently, 10 μL of CCK-8 reagent was added to each well, mixed gently, and incubated for 4 hours under standard conditions. The absorbance at 450 nm was then measured for each well.

### Transwell assay

Cell migration of HGC-27 cells was assessed using the Transwell assay. Briefly, cells were cultured in a 24-well Boyden Chamber (1×10^5^ cells per well, 8 μm pore size, NEST, China). After 24 hours’ treatment with 10 µg/mL hr MFAP5, migrated cells on the inserts were stained with crystal violet and quantified under an optical microscope (Leica, Germany).

### Western blotting

The cells were lysed in RIPA buffer (Solarbio, China) with protease inhibitors (MCE, USA) to extract total protein content. The proteins were boiled in SDS sample buffer, separated on SDS-PAGE, and transferred to PVDF membranes (Millipore, USA). After blocking with 5% skim milk, the membranes were incubated overnight at 4°C with primary antibodies against anti- anti-Notch2 (ab245325, Abcam, UK), and anti-HEY1 (DF12076, Affinity, China). Subsequently, secondary antibodies were applied for 2 hours before visualizing the bands using ECL detection reagent (Vazyme, China).

### Statistical analysis

All the detailed information on applied software or packages is listed in **Supplementary Table 4**. The statistical data analyzed in this study were all performed in R studio (4.1.3). The log-rank test was used to assess the significance of Kaplan-Meier survival curves. Group comparisons were made using Student’s t-test, Wilcoxon rank-sum test, and Kruskal-Wallis test. Additionally, Fisher’s exact test and the Chi-square test were employed to evaluate associations among categorical variables. For visualization purposes, we utilized the following packages: Seurat, ggplot2, ggrepel, ggpubr, ComplexHeatmap, CellChat, corrplot, and survminer. The (adjust) p-value<0.05 was considered as statistically significant.

## Results

### Landscape of fibroblast phenotypes in gastric cancer

**Figure 1:**
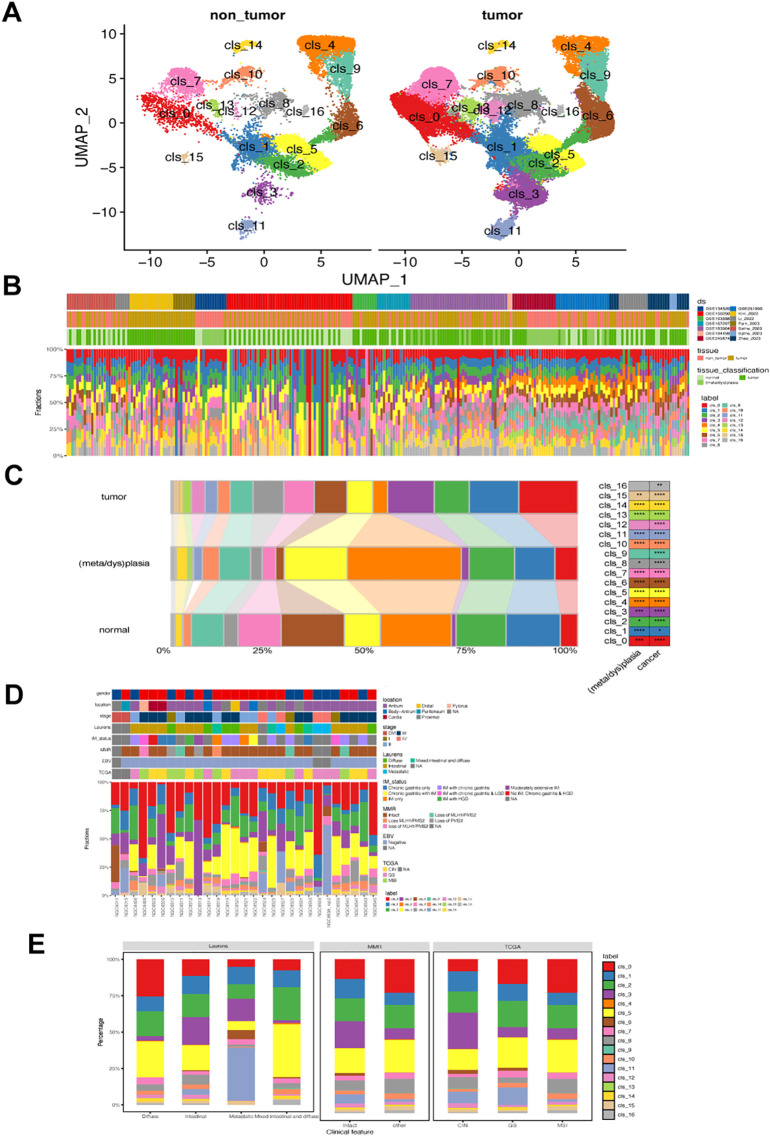
Landscape of fibroblast phenotypes in gastric cancer (GC). **(A)** UMAP projections of 63,955 fibroblasts in non-tumor (left) and GC tumor tissues (right), colored by cluster identify. **(B)** Distribution of each fibroblast cluster among samples from 14 single-cell RNA-seq datasets. Ds, datasets **(C)** Bar plots showing the ratio of each fibroblast cluster in normal, metaplasia/dysplasia, and tumor tissues. **(D)** Characterization of the prevalence of fibroblast clusters in GC samples from the GSE183904 dataset. **(E)** Bar plot displaying differences in prevalence of various fibroblast clusters among different Lauren’s, MMR, and TCGA subtypes in GSE183904 dataset. The classification information was adapted from the original study. MMR, mismatch repair; CIN, chromosomal instability; GS, genomically stable; MSI, microsatellite instability; TCGA, The Cancer Genome Atlas.

We analyzed fibroblast diversity in gastric cancer by compiling and standardizing data from fourteen datasets, identifying 63,955 fibroblast cells grouped into 17 subsets (named clusters 0-16, **Figure 1A**). Cluster 0-9 were the predominant fibroblasts in GC, with cluster 10-16 showing relative lower prevalence (**Figure 1B**). These fibroblast clusters were found in both tumor and non-tumor tissues but with varying ratios among normal, metaplasia/dysplasia, and tumoral tissues (**Figures 1B, C**). Notably, GC exhibited a distinct composition of fibroblasts compared to normal and metaplasia/dysplasia tissues, with increased levels of clusters 0, 8, 13, and 15 (**Figures 1B, C**). Specific fibroblast subsets correlated with histological and molecular classifications in tumor samples (**Figures 1D, E**); for instance, metastatic subtype had lower levels of clusters 5, 10, but higher levels of cluster 11. The dMMR subtype showed reduced prevalence of clusters 0 and 5 but increased levels of clusters 3 and 11. Variations were also observed in the abundance of clusters 0,3,6 and 11 among TCGA-CIN, GS and MSI subtypes (**Figure 1E**).

### Correlation between identified and previously-defined fibroblast subsets

**Figure 2:**
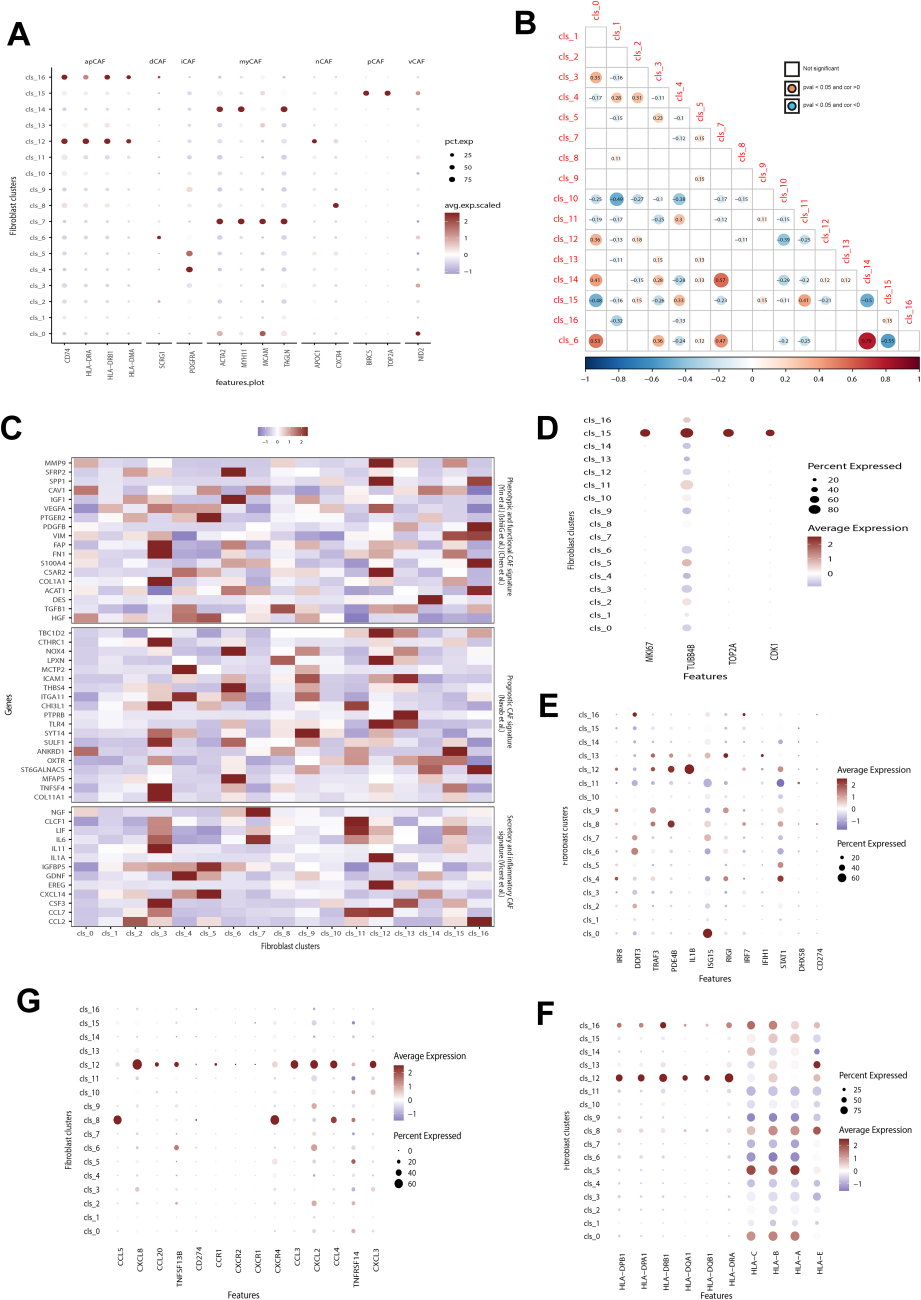
Correlation between identified and previously-defined fibroblast subsets. **(A)** Analyzing the expression of hallmark genes for previously defined fibroblast subsets in identified clusters. Dot color and size indicate average expression level and percentage, respectively. apCAF, antigen-presenting CAF; dCAF, divergent CAF; iCAF, inflammatory CAF; myCAF, Myofibroblastic CAF; nCAF, normal fibroblast; pCAF, proliferatory CAF; vCAF, vascular CAFs. **(B)** Exploring the correlation among identified fibroblast clusters. The red dot shows a positive correlation between two clusters, while the blue dot indicates a negative correlation. The dot with color indicates a p value below 0.05. **(C)** Evaluation the expression of hallmark genes associated with known prognostic, phenotypic, functional, secretory, and inflammatory CAFs signatures. **(D–G)** The difference in the expression of proliferation-related **(D)**, interferon-related genes **(E)**, cytokines **(F)**, and MHC class II molecules **(G)** among different CAFs fibroblasts. Dot color and size indicate average expression level and percentage, respectively. CAFs, cancer-associated fibroblast.

We compared our identified clusters with previously classified fibroblast subsets and found distinct features in our subsets compared to known CAF subsets, such as iCAF and mCAF (**Figure 2A**). While some of our subsets showed associations with previously identified subpopulations, others exhibited unique characteristics. For instance, cluster 12 and 16 both expressed apCAF-related markers like CD74, HLA-DRA, HLA-DRB1, and HLA-DMA. However, cluster 12 also exhibited elevated expression of CXCL2 and APOC1, which markers associated with iCAF and nCAF, respectively. Additionally, several clusters, such as cluster 6, showed high expression levels of iCAF-associated markers, but also overexpressed markers from other CAF subpopulations. Despite most clusters showing overexpression of known CAF markers, a subset of fibroblasts (clusters 1, 3, 9, 10, and 13) could not be classified into any known CAF subsets. Cluster 13 also exhibited Endothelial-related marker expression. We successfully distinguished endothelial cells from endo-fibroblasts (cluster 13) and other fibroblasts in GSE249874 (**Supplementary Figure 2**), suggesting they may have undergone endothelial-to-mesenchymal transition (EndMT) (26). These discrepancies underscore the need for more refined fibroblast subsets in GC.

The fibroblast clusters in **Figure 2B** showed varying relationships of coexistence and exclusion. For instance, cluster 10 had a significant exclusion correlation with clusters 1 and 4, while cluster 6 was strongly correlated with clusters 14, 0, and 7 but excluded from cluster 15. A comparison of the expression levels of fibroblast subsets was conducted based on genes associated with prognostic, phenotypic, functional, secretory, and inflammatory CAF signatures (**Figure 2C**). These subsets displayed notable heterogeneity in these signatures. Some subtypes like clusters 0, 1, and 10 showed weak correlations to these features. In contrast, clusters 3,6,9,12 and16 exhibited strong associations with these CAF signatures. Notably among them were clusters3, 6 and9 which are relevant for prognosis in GC patients.

Biological features such as cell cycle were then analyzed for the identified fibroblast subsets (**Figures 2D–G**). Cluster15 showed significantly higher expression of cell cycle genes (incluging MI67, UBB4B, TOP2A, and CDK1) compared to all other subsets indicating increased proliferation activity (**Figure 2D**), aligning with its similarity to pCAF. On the other hand, Custer 11 had low expression levels of interferon-related genes especially ISG15 and STAT1 whereas Cluster12 demonstrated significantly higher activity in interferon-related pathways notably having the highest IL-1B expression level (**Figure 2E**). Additionally Clusters12 along with Clusters16,8, 5 and 0 also exhibited significantly higher expressions levels in both cytokines (**Figure 2F**) and MHC class II molecules (**Figure 2G**).

### Biological and metabolism feature of fibroblast subsets

**Figure 3:**
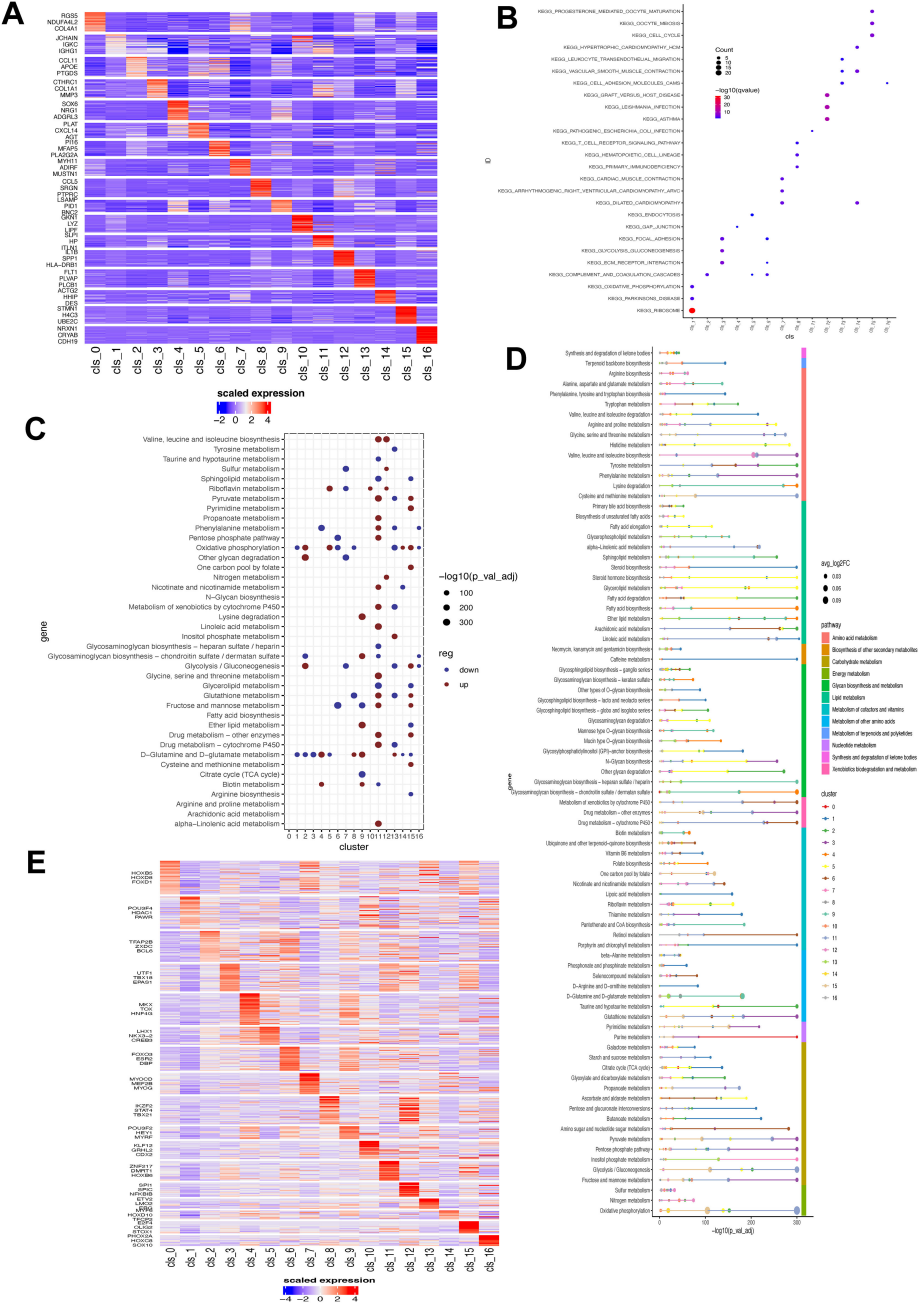
Biological and metabolism feature associated with fibroblast clusters. **(A)** Heatmap showing the expression level of top three DEGs in each fibroblast cluster. **(B)** Enrichment of biological pathways of DEGs of each fibroblast cluster. The size of dot indicates the count of enriched DEGs and its color shows the statistical significance. **(C)** ScMetablism analysis showing the activity of different metabolism pathways in each fibroblast cluster. The red dot represents upregulation, and the blue dot signifies downregulation. The dot size shows the statistical significance. **(D)** Metabolism pathway activity of fibroblast subsets. **(E)** Transcription factor expression heatmap in each fibroblast subset. DEG, differentially-expressed gene.

To uncover the biological significance of each cluster, we performed enrichment analysis on their DEGs (**Figure 3A**). Then, the top 50 upregulated genes in each cluster were utilized to analyze the biological pathways. As illustrated in the **Figure 3B**, all identified fibroblasts had unique enrichment of biological pathways. For instance, cluster 1 showed a significant association with ribosome and cluster 12 showed correlation with immune-related disease. Similarly, these fibroblast subsets showed great heterogeneity in metabolism (**Figure 3C**). For example, cluster 11 had the highest activity in the majority of metabolism pathways, and cluster 6, 13 had the most down-regulated metabolism pathways. On the other hand, oxidative phosphorylation, and D glutamine/glutamate metabolism pathways were the most altered in those subsets. The results revealed significant heterogeneity in various metabolic pathways, such as amino acid, energy, and lipid metabolism (**Figure 3D**), suggesting potential metabolic coordination among tumor fibroblasts. Additionally, we examined the differences in transcription factor (TF) expression across these fibroblast clusters and observed distinct upregulation of TFs in each cluster (**Figure 3E**). Notably, Cluster 1 exhibited downregulation of most TFs except for POUSF4, HDAC1, and PAWR. Cluster 12 showed an increase in SPI1, SPIC, and NFKB1B expression, and cluster 8 upregulated IKZF2, STAT4, and TBX21.

### Differentiation state of fibroblast subsets

**Figure 4:**
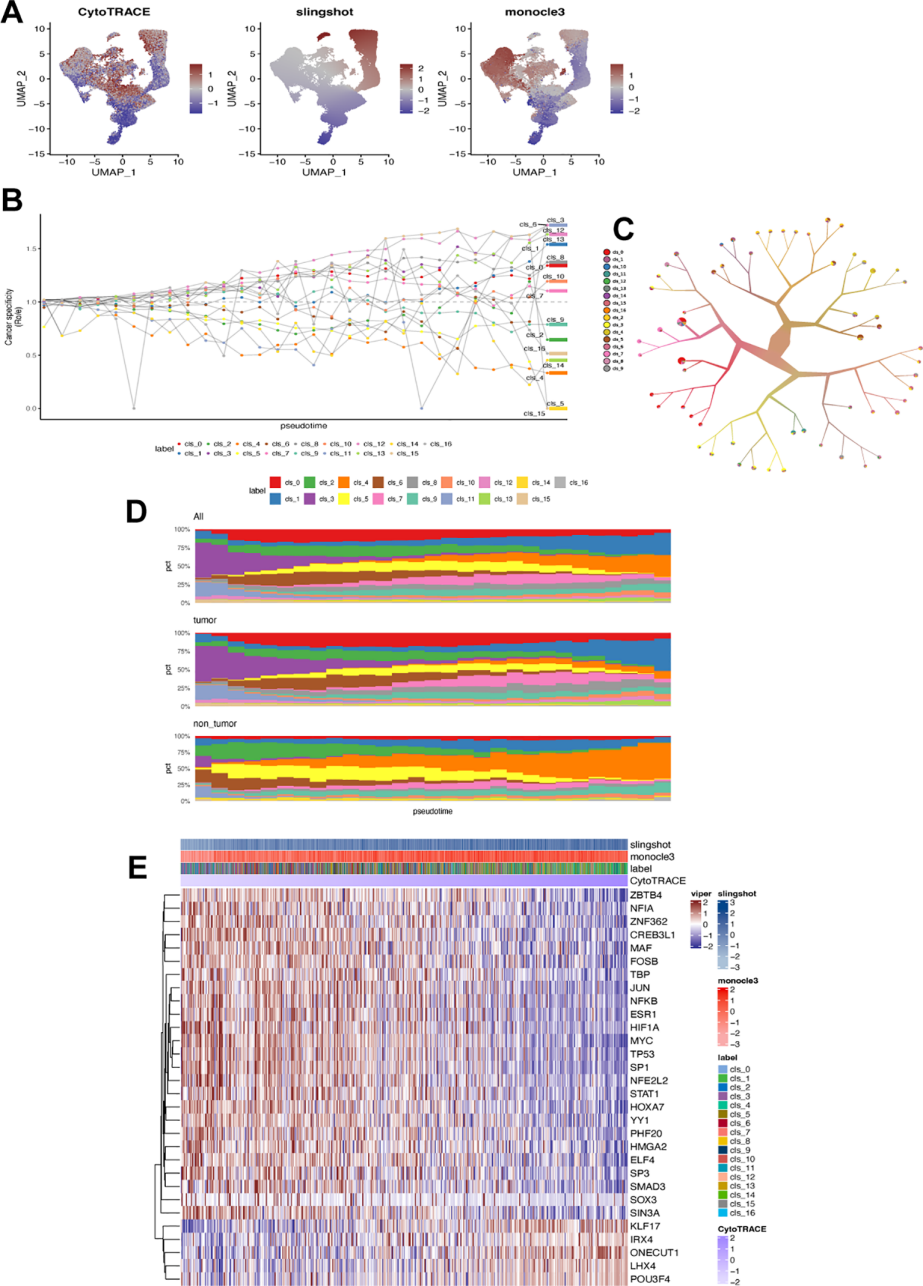
Differentiation state of each fibroblast cluster. **(A)** UMAP plot displaying estimated differentiation state by CytoTRACE, monocle3, and Slingshot, respectively. **(B)** Fibroblast ratio of observed to expected cell numbers (Ro/e). **(C)** Fibroblast clustering based on tree structure using the TooManyCells algorithm. **(D)** Ranking fibroblast subsets by Ro/e and differentiation state (pseudotime) in all samples, tumor samples, and non-tumor samples. **(E)** Heatmap showing correlation between transcription factors and differentiation state in each CAFs cluster (label).

We used monocle3, CytoTRACE, and Slingshot separately to analyze fibroblast trajectories based on pseudotime (**Figure 4A**). Cluster 3 may have persisted at the endpoint due to its specificity to tumors and pseudotime values (**Figure 4B**). To understand the maturation state divergence of identified fibroblast subsets, we examined their branching patterns using a tree structure (**Figure 4C**), revealing a continuous differentiation state with cluster 7 having the highest pseudotime value. These subsets were distributed differently between tumor and normal gastric tissues: clusters 0, 3, 11, 12, and 15 were more abundant in tumors while clusters 4, 5, and 15 were prevalent in normal tissues (**Figure 4D**). Analyzing changes over pseudotime showed that as it increased, clusters 3, 12, 13 and 1 became enriched in tumor tissues while cluster4 was more prevalent in normal tissues. We also investigated how these subsets differentiated by analyzing the correlation between transcription factors and pseudotime. The results indicated that multiple TFs played a role; particularly MYC ONECUT1 POU3F4 influenced the differentiation state of fibroblast subsets (**Figure 4E**). The key feature of all the identified fibroblast cluster in GC was summarized in **Supplementary Table 6**.

### Cluster 6 was associated with worse clinical outcomes

**Figure 5:**
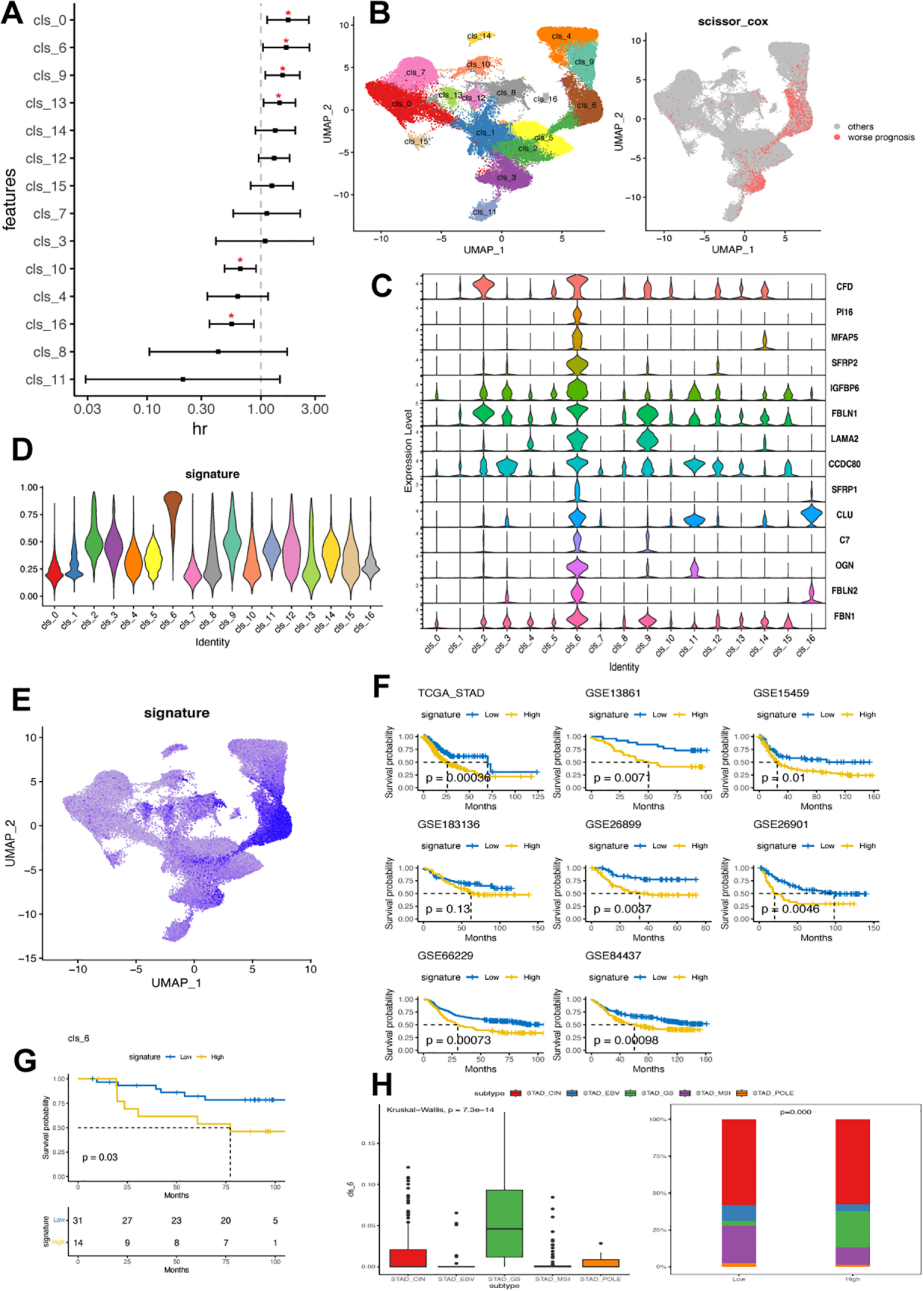
Cluster 6 of CAFs was associated with worse clinical outcomes in GC. **(A)** Forest plot illustrating the correlation between identified fibroblast cluster and overall survival in TCGA-STAD dataset. The x-axis shows the hazard ratio (HR), with a dotted vertical line at HR value of 1, while the y-axis represents each fibroblast cluster. **(B)** Linking fibroblast composition to poorer prognosis using SCISSOR algorithm. The red dots in the right UMAP plot indicate the cells associated with worse prognosis in the TCGA-STAD dataset. **(C)** Violin plots displaying the expression profile of 14 hallmark genes associated with cluster 6 in identified fibroblast clusters. **(D)** Discrepancy in cluster 6 signature value across fibroblast clusters. **(E)** UMAP plot demonstrating cellular composition based on cluster 6 signature value in GC scRNA-seq dataset. **(F)** Kaplan-Meier plots comparing high and low levels of cluster 6 signature among patients in eight GC bulk RNA datasets. **(G)** Kaplan-Meier plot comparing high and low levels of cluster 6 signature among patients in a local GC cohort. **(H)** Variation in cluster 6 signature value among patients with different molecular subtypes (TCGA) within the TCGA-STAD cohort. EBV, Epstein-Barr virus; MSI, microsatellite instable; GS, genomically stable; CIN, chromosomal instability; GC, gastric cancer; TCGA, The Cancer Genome Atlas; STAD, stomach adenocarcinoma.

To gain a deeper understanding of the biological and clinical functions of identified fibroblast clusters, we selected the top 50 genes with high specific expression in each cluster as its signature, excluding mitochondrial and ribosomal genes. The AUCell method was then utilized to assess this signature in single-cell data clusters. Analyzing the relationship between fibroblast subsets and prognosis in GC patients, we examined their signatures in the TCGA-STAD dataset. Clusters 0, 6, 9, and 13 were significantly associated with poorer survival outcomes while clusters 16 and 10 showed a favorable correlation (**Figure 5A**). To validate these associations further, we employed the SCISSOR method (**Figure 5B**), which indicated that cells linked to worse prognosis were predominantly found in cluster 6. Consequently, our subsequent study focused on investigating the characteristics of cluster 6 in GC.

A total of 1260 DEGs were identified between cluster 6 and the other clusters. These DEGs were primarily enriched in DNA repair, epithelial-mesenchymal transition, cell cycle, hypoxia, complement activation, regulation of peptidase activity, collagen containing extracellular matrix and immunology-related pathways (**Supplementary Table 6**). Then, the expression signature of cluster 6 was defined by 14 identified DEGs, including CFD, PI16, MFAP5, SFRP2, IGFBP6, FBLN1, LAMA2, CCDC80, SFRP1, CLU, C7, OGN, FBL2 and FBN1 (**Figure 5C**). Notably, PI16, MFAP5 and SFRP2 were exclusively expressed in cluster 6. Cluster 6 exhibited the highest signature scores consistent with the observation that cells showing a positive signature were predominantly found in cluster 6 (**Figures 5D, E**).

We confirmed a correlation between cluster 6 and poor clinical outcomes in seven additional GC datasets. The results consistently showed that samples with high levels of cluster 6 had significantly worse clinical outcomes (**Figure 5F**). Our local GC cohort also indicated that patients with high cluster 6 had significantly shorter overall survival (**Figure 5G**). Interestingly, samples with high cluster 6 were mainly found in the GS subtype, which had lower frequencies of EBV, MSI, and POLE subtypes (**Figure 5H**). Furthermore, cluster 6 sig did not show significant correlations with clinical traits but emerged as an independent prognostic feature in GC (**Supplementary Table 7**).

### Analysis of cluster 6 fibroblast cell-cell communication

**Figure 6:**
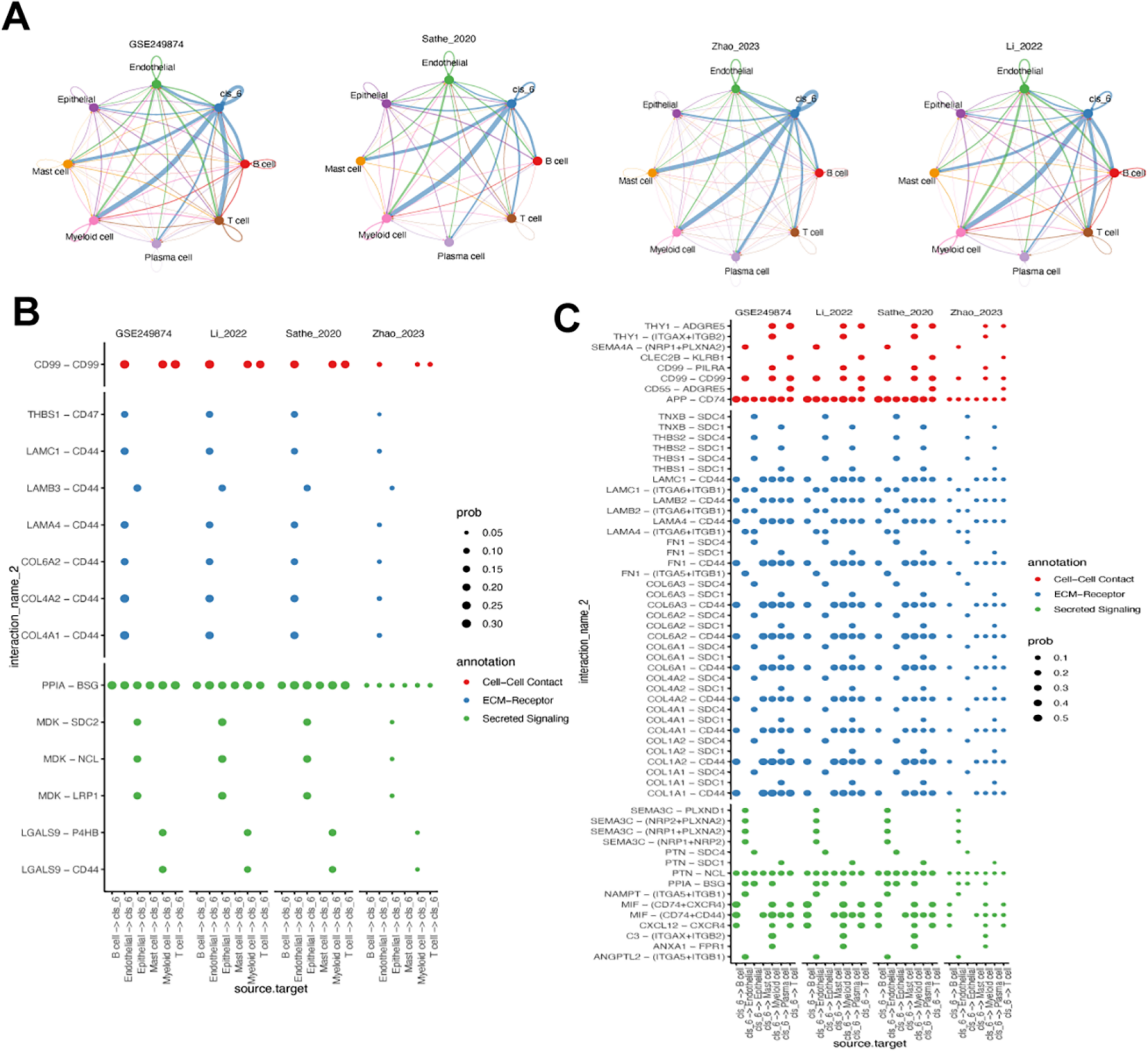
Analysis of cluster 6 fibroblast-related cell-cell communication. **(A)** Circos plot of the cellular crosstalk of cluster 6 fibroblasts toward the major immune cells in four gastric cancer scRNA-seq datasets. **(B)** Immune cell - cluster 6 fibroblast crosstalk in cell-cell contact, ECM-receptor and secreted signaling pathways. **(C)** Cluster 6 fibroblast- immune cells crosstalk in cell-cell contact, ECM-receptor and secreted signaling pathways.

We analyzed the cell-cell communication of cluster 6 using the cellChat ligand-receptor complex databases. Results from four scRNA-seq datasets showed that cluster 6 fibroblasts primarily interacted with myeloid cells, followed by mast cells and T cells (**Figure 6A**). Comparing the crosstalk between immune cell and cluster 6 fibroblast (**Figure 6B**) with the Cluster 6 fibroblast- immune cells crosstalk (**Figure 6C**), it is evident that cluster 6 fibroblasts play a significant role in these interactions. All immune cells were able to contact cluster 6 through PPIA-BSG secreted signaling (**Figure 6B**). Additionally, B cells, myeloid cells, and T cells interacted with cluster 6 via CD99-CD99. Conversely, cluster 6 engaged with other immune cells through APP-CD74, PTN-NCL, and CD44-based contacts (**Figure 6C**). All in together, the results potentially implicating cluster 6 -myeloid cell interaction may contribute to the worse prognosis in GC.

### MFAP5 expressed in fibroblast associated with worse prognosis

**Figure 7:**
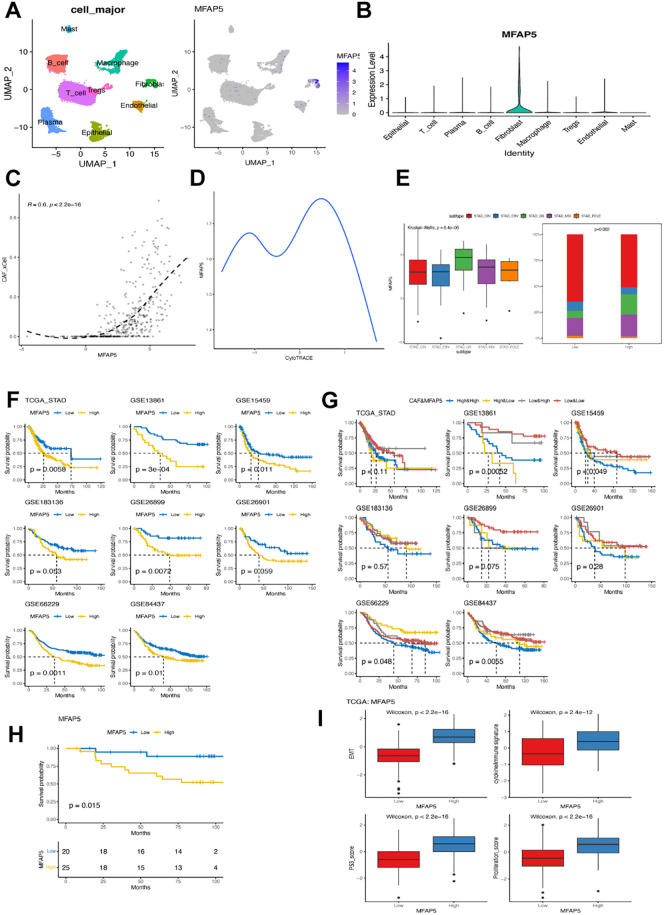
MFAP5 expression in fibroblast associated with worse prognosis in GC. **(A)** UMAP plot illustrating the distribution of MFAP5 expression in major cell types within the GSE167297 dataset. **(B)** Bar plot displaying variations in MFAP5 levels across different major cell types in the GSE167297 dataset. **(C)** Examining the correlation between MFAP5 expression and CAFs abundance in the TCGA-STAD dataset. **(D)** Analyzing MFAP5 expression alongside estimated differentiation state using CytoTRACE. **(E)** Contrasting levels of MFAP5 expression among GC patients with diverse molecular subtypes from the TCGA-STAD dataset. **(F)** Kaplan-Meier analysis comparing high and low levels of MFAP5 among patients across eight gastric cancer datasets. **(G)** Kaplan-Meier analysis contrasting high and low levels of MFAP5 among patients within a local gastric cancer cohort. **(H)** Survival comparison among patients stratified by both MFAP5 and fibroblast levels across eight gastric cancer datasets using Kaplan-Meier plots. **(I)** Investigating differences in cytokine/immune signature, p53 score, EMT score, and proliferation score between patients with high versus low levels of MFAP5 expression.

As MFAP5 showed the least statistical significance among cluster 6-related DEGs, we delved into its biological and clinical characteristics in GC. We found that MFAP5 was present not only in tumor tissues but also in fibroblasts from non-tumor tissues (**Supplementary Figure 3**), with comparable expression levels between normal and tumor tissues, suggesting a unique role for MFAP5 or cluster 6 within both microenvironments. Our investigation revealed predominant expression of MFAP5 in fibroblasts (**Figures 7A, B**) and a strong correlation between MFAP5 expression and CAF levels using bulk RNA sequencing data (**Figure 7C**). Pseudotime analysis hinted at a decreasing trend of MFAP5 during fibroblast evolution/transformation process (**Figure 7D**), aligning with significantly higher levels of MFAP5 expression among GS subtypes within cluster 6 fibroblast samples (**Figure 7E**). Comparing different GC molecular subtypes, we observed that the high-MFAP group had a significantly higher ratio of GS subtype but lower ratios of CIN, EBV, and POLE subtypes (**Figure 7E**).

Further analysis focused on exploring correlations between MFAP5 expressions and clinical features. Significant associations were found between MFAP5 expressions with tumor grade and stage but not lymph node or distant metastasis stages. Across eight analyzed GC datasets, high-MFAP5 expressions were consistently linked to worse prognoses; patients with high-MFAP5 expressions displayed inferior clinical outcomes consistently across datasets (**Figures 7F, G**, respectively). Notably, patients showing both high-CAFs and high-MFAP5 expressions had notably shorter overall survival rates compared to other groups. In our local cohort, samples with high MFAP5 expression also had significantly worse prognosis (**Figure 7H**). Additionally, elevated cytokine/immune signatures along with increased p53 score EMT score proliferation scores within the high-MFAP5 group could contribute towards their association with poor clinical outcomes seen in GC cases (**Figure 7I**).

### Cluster 6 and MFAP5 were related to immunotherapy response in GC

**Figure 8:**
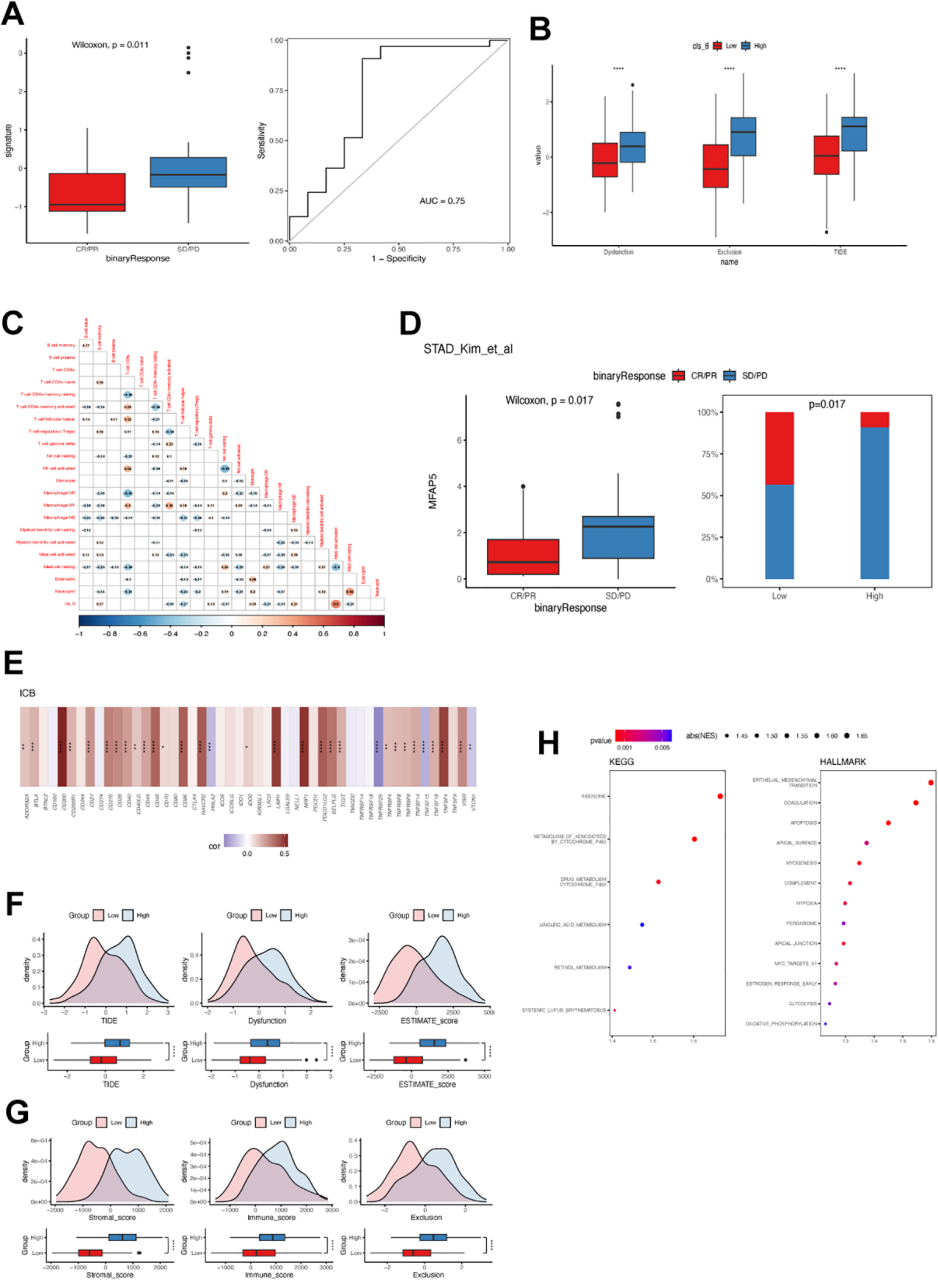
Cluster 6 and MFAP5 were related to immunotherapy response in GC. **(A)** Differences in cluster 6 fibroblast levels among GC patients with or without objective response to immunotherapy. ROC curve demonstrates the predictive accuracy of cluster 6 fibroblast. **(B)** Difference in TIDE value between GC patients with high and low cluster 6 fibroblast. **(C)** Correlation between cluster 6 and tumor-infiltrated immune cells in TCGA-STAD cohort. **(D)** Differences in MFAP5 expression levels among GC patients with or without objective response to immunotherapy. **(E)** Correlation between MFAP5 and immune checkpoint expression in TCGA-STAD cohort. **(F)** Difference in TIDE value between GC patients with high and low MFAP5. **(G)** Differences in immune and stromal scores between GC patients with high and low MFAP5. **(H)** Enrichment of biological pathways related to MFAP5. *p<0.05; **p<0.01; ***p<0.001; ****p<0.0001.

Furthermore, cluster 6 fibroblast expression was associated with a poor response to immunotherapy in GC. Patients who responded to pembrolizumab as salvage treatment had significantly lower abundance of cluster 6 fibroblasts (**Figure 8A**). Similarly, GC patients with high levels of cluster 6 fibroblasts exhibited significantly higher T cell dysfunction, exclusion, and TIDE score, which supported the negative association between cluster 6 fibroblast and immunotherapy sensitivity (**Figure 8B**). The correlation analysis revealed that cluster 6 fibroblasts were strongly linked to activated mast cells and M2 macrophages (**Figure 8C**). Conversely, it had a weak but significant negative correlation with T CD4+ memory activated cells and M0/M1 macrophages. Consistently, individuals with low MFAP5 expression were predominantly immunotherapy responders (**Figure 8D**). Intriguingly, MFAP5 showed a significant positive correlation with most immune checkpoints including CD274 but had negative correlations with HHLA2, TNFRSF25, TNFRSF15, and VCTN1 (**Figure 8E**). While the high-MFAP5 group displayed higher immune and stromal scores in GC samples, its association with T cell dysfunction and exclusion may explain its negative correlation with immunotherapy response (**Figures 8F, G**). Additionally, MFAP5 upregulated EMT pathways along with hypoxia and MYC pathways in GC samples which could contribute to reduced sensitivity to immunotherapy (**Figure 8H**).

### MFAP5 promotes tumor cell proliferation and migration in GC

**Figure 9:**
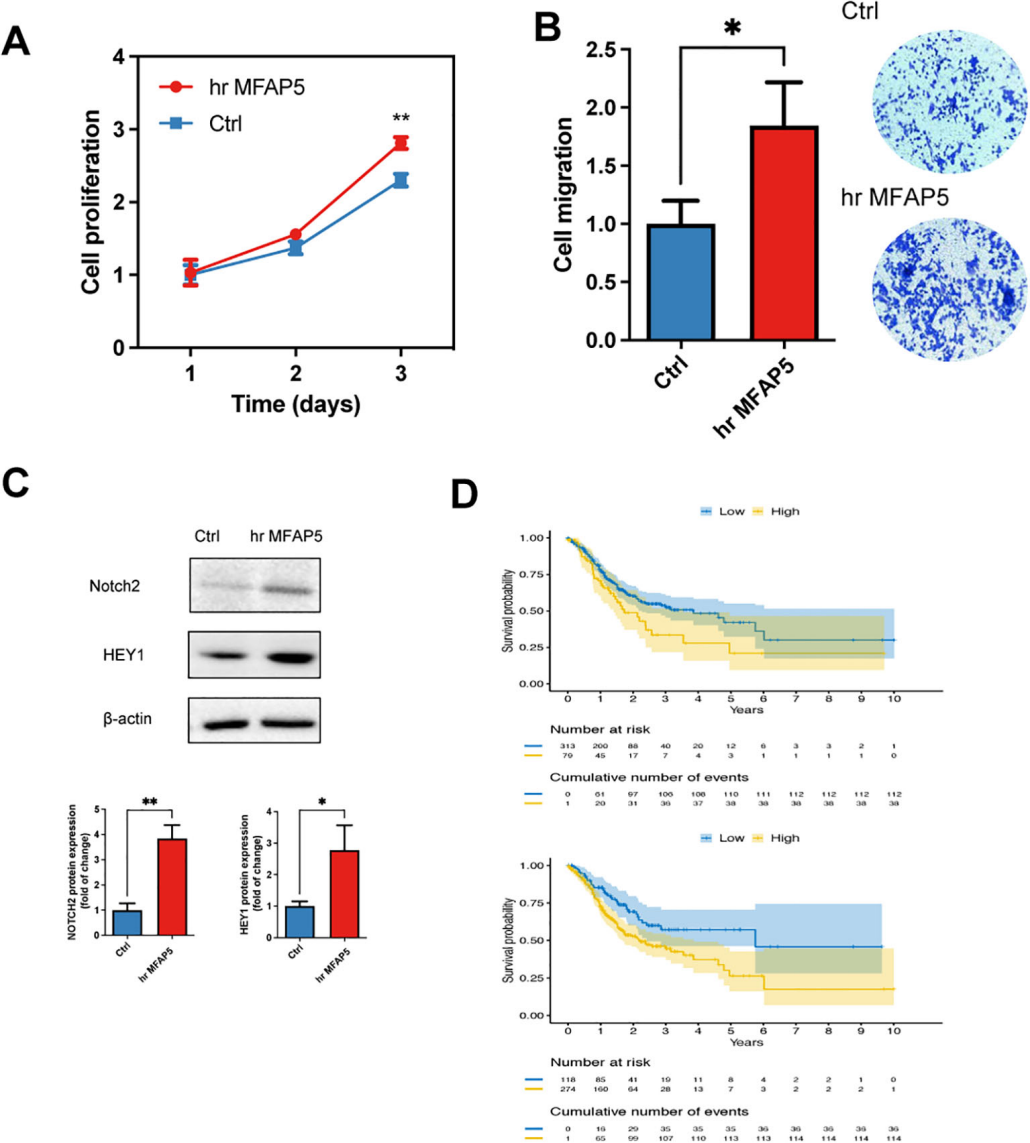
MFAP5 promotes the GC cell proliferation and migration. **(A)** Assessment of cell proliferation in hr MFAP5-treated HGC-27 cells. **(B)** Evaluation of cell migration in hr MFAP5-treated HGC-27 cells. **(C)** Western blotting to examine Notch2 and HEY1 levels in hr MFAP5-treated HGC-27 cells. **(D)** Kaplan-Meier analysis comparing high and low expression levels of NOTCH2 or HEY1 among patients in the TCGA-STAD cohort. hr MFAP5, human recombinant MFAP5. *p<0.05; **p<0.01.

Next, we evaluated the effect of adding MFAP5 in the GC cells *in vitro*. Adding of MFAP5 significantly stimulated the proliferation and migration of HGC-27 cells (**Figures 9A, B**). To delve deeper into the MFAP5-regulated signaling pathway, we conducted a literature review and found that MFAP5 might activate the Notch2 and or HEY1 in other cancer type and Notch2 has been recognized as an oncogene that enhances invasion in GC. We observed higher levels of Notch2 and HEY1 in HGC-27 cells treated with MFAP5 compared to the control group (**Figure 9C**). Gastric cancer patients exhibiting high NOTCH2 or HEY1 expression had significantly poorer prognosis than those with lower levels, respectively (**Figure 9D**). Overall, these findings suggested a potential mechanism that MFAP5 promoted gastric cancer progression through the MFAP5/Notch2/HEY1 signaling axis.

## Discussion

In our current study, we created a comprehensive atlas of single-cell transcriptomes in GC by combining 14 datasets comprising 63,955 fibroblasts from 905,186 cells. We offer a detailed overview of the fibroblast landscape in GC, identifying 17 distinct subsets. Among these, only eight showed significant expression of known CAF biomarkers; the remaining nine subsets did not fit into existing CAF categories. In addition to differences in gene expression, these fibroblast subsets also exhibited variations in metabolism, cell interactions, and clinical implications. Notably, cluster 6 stood out for its high expression levels of SCRG1 (a marker for developmental CAFs), as well as CFD, C3, and CXCL2 (markers for inflammatory CAFs). This cluster has the potential to refine tumor classification in GC prognosis and response to ICIs. Developmental CAFs share gene expression patterns with GC tumor cells and undergo epithelial-mesenchymal transition (EMT) (27); however their specific role in GC remains unclear at this time.

Of particular interest for cancer immunotherapy is the cluster 6 with an immunosuppressive- feature in GC, associating with not only the inferior prognosis but also the primary resistance to ICIs. This correlation between cluster 6 and ICIs insensitivity may be attributed by its contact with tumor macrophage (especially M2 macrophages) and resist the infiltration of T cells. Previous study has found that three groups of cancer embryonic cells, including POSTN+ CAF, FOLR2+ TAM, and PLVAP+ EC, have close cellular communication connections in hepatocellular carcinoma (28). This particular type of CAFs, known as POSTN+ CAFs, play a significant role in shaping the environment for “cancer-embryo” reprogramming. They act as a central communication hub by releasing different molecules like CXCL12, CXCL16, and IL34. However, despite cluster 6 showing increased levels of CXCL12, their POSTN levels were low. This suggests that there is a distinct mechanism at work in cluster 6 when it comes to shaping the immunosuppressive TME.

Targeting the stromal environment offers hope for improving therapeutic response, and our study reveals that the biological and clinical function of MFAP5, a specific marker of cluster 6. Although the function of MFAP5 has not been well established in both CAFs and GC, its oncogenic role and negative impact on patients’ clinical outcomes have been widely supported in other types of cancer (29–33). However, they mainly explored the function of MFAP5 in tumor but not specifically in CAFs with only the exception of Duan et al. to date (31). They found that inhibiting MFAP5-high CAFs enhances the effectiveness of gemcitabine-based chemotherapy and PD-L1-based immunotherapy, which was in consistent with our findings (34). The MFAP5-related ICI resistance was based on the transduction of MFAP5/RCN2/ERK/STAT1 pathway, regulating angiogenesis, hyaluronic acid levels, collagen deposition, and infiltration of cytotoxic T cells. Blocking CXCL10 with AMG487 has the potential to reverse the pro-tumor effect and enhance immunotherapeutic efficacy when combined with anti-PD-L1 antibody. Given the correlation between MFAP5 and ICI insensitivity, targeting MFAP5 could be a promising therapy to improve immunochemotherapy effects in gastric cancer by reshaping the desmoplastic and immunosuppressive microenvironment. On the other hand, we found the association between MFAP5 and NOTCH pathway, indicating that Notch inhibitors may be the promising regimens to combine with ICI in treating MFAP5-high GC. Previous studies in other studies have supported the connection between MFAP5/CAFs and Notch family genes (29, 30, 35). Pharmaceutical therapy targeted at Notch pathways, including γ-secretase inhibitors, ADAM inhibitors, antibodies targeting Notch receptors or ligands and Notch transcription complex inhibitors are currently under clinical studies (36).

Several challenges and limitations of the current study require attention. Firstly, extending our analysis to include scRNA-seq or multiplex immunofluorescence analysis using local GC samples is necessary due to the primary use of public datasets. Secondly, the sample size for RNA-seq in our local cohort was limited to 45 patients; thus, expanding this from our center is essential in future studies. Thirdly, despite validating our results in various external scRNA-seq cohorts and attempting to address batch effects across different datasets, these effects must still be considered for confirming findings. Additionally, as there are few GC patients undergoing immunotherapy currently and since our conclusions rely on transcriptomic data from public databases, validation of the relationship between cluster 6 or MFAP5 expression and immunotherapy responsiveness in an immunotherapy cohort is crucial going forward. Finally, due to challenges in obtaining CAFs from fresh tumor samples, additional experiments both *in vivo* and *in vitro* are required to determine MFAP5 expression profiles in tumor cells and CAFs. In the current study, we have identified an association between cluster 6 and MFAP5 with poor outcomes and immunotherapy resistance. However, further research is needed to gain detailed mechanistic insights into how these factors contribute to the immunosuppressive microenvironment. Additionally, it is important to investigate the other clusters apart from cluster 6 to better understand their biology and clinical features.

In conclusion, we present a high-resolution GC fibroblast atlas. Each of the 17 identified fibroblast clusters offers opportunities for gaining deeper biological insights into the relationship between fibroblasts and GC development. Specifically, cluster 6 and its specific marker MFAP5 could serve as prognostic factors in GC and provide a basis for personalized therapeutic combinations to overcome primary resistance to ICIs.

## Data Availability

The datasets presented in this study can be found in National Genomics Data Center, China National Center for Bioinformation / Beijing Institute of Genomics, Chinese Academy of Sciences (https://ngdc.cncb.ac.cn/gsa-human.) under the accession number of HRA008925.
