# Supplementary material for: High-resolution subtyping of fibroblasts in gastric cancer reveals diversity among fibroblast subsets and an association between the MFAP5-fibroblast subset and immunotherapy
Source: Front Immunol. 2024 Oct 25;15:1446613. doi: 10.3389/fimmu.2024.1446613 (PMC11543424; doi:10.3389/fimmu.2024.1446613)

Supplementary figure 1: schematic diagram of study design

Supplementary figure 2: The expression profile of endothelial and fibroblast markers in endothelial, endo-fibroblast (cluster 13) and other fibroblast from the GSE249874.

Supplementary figure 3: UMAP plots showing the distribution of MFAP5 expressed fibroblast in non-tumor tissues.

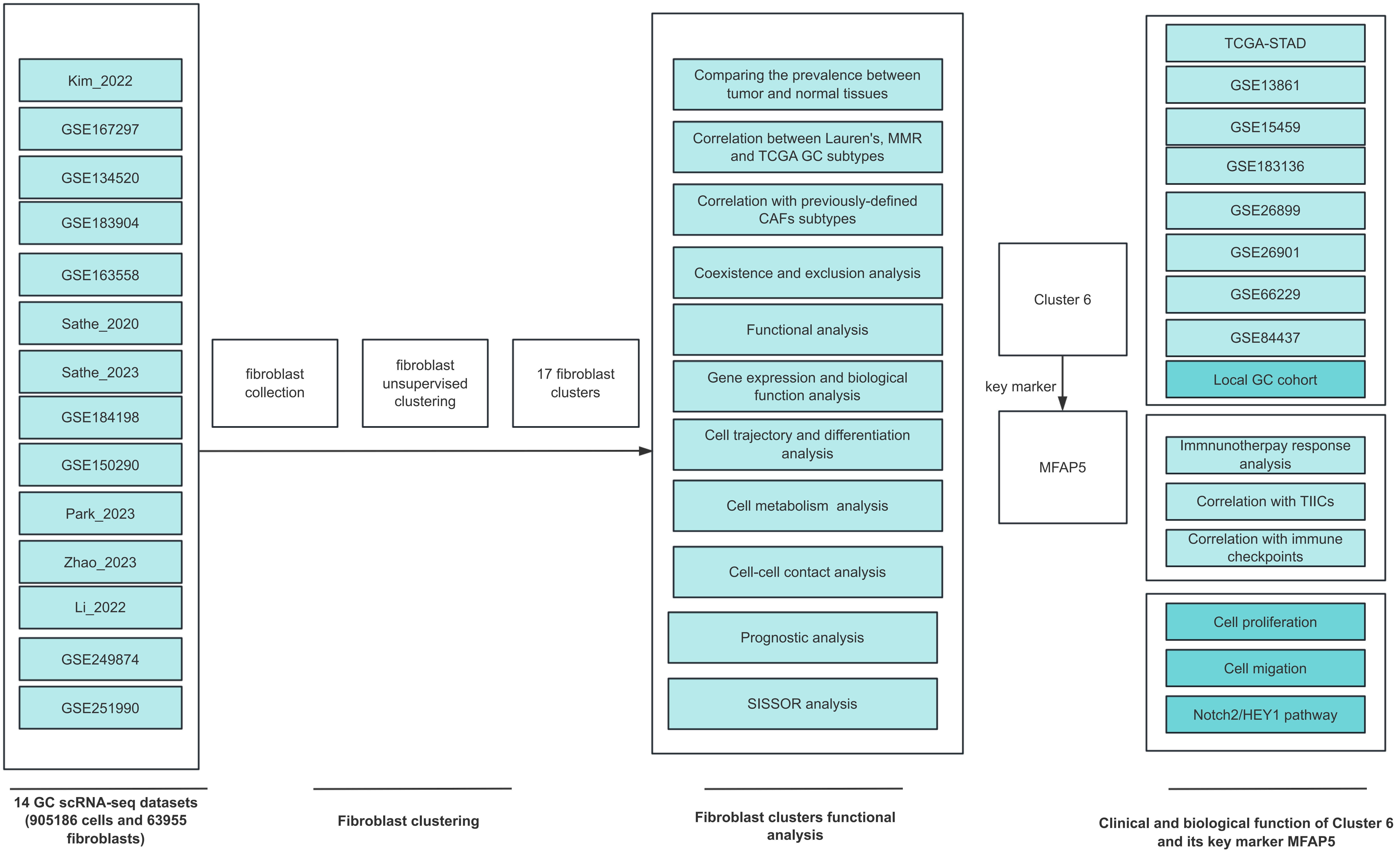
Kim_2022
GSE167297
GSE134520
GSE183904
GSE163558
Sathe_2020
Sathe_2023
GSE184198
GSE150290
Park_2023
Zhao_2023
Li_2022
GSE249874
GSE251990
fibroblast collection
fibroblast unsupervised clustering
17 fibroblast clusters
Comparing the prevalence between tumor and normal tissues
Correlation between Lauren's, MMR and TCGA GC subtypes
Correlation with previously-defined CAFs subtypes
Coexistence and exclusion analysis
Functional analysis
Gene expression and biological function analysis
Cell trajectory and differentiation analysis
Cell metabolism analysis
Cell-cell contact analysis
Prognostic analysis
SISSOR analysis
Cluster 6
key marker
MFAP5
TCGA-STAD
GSE13861
GSE15459
GSE183136
GSE26899
GSE26901
GSE66229
GSE84437
Local GC cohort
Immnunotherpay response analysis
Correlation with TIICs
Correlation with immune checkpoints
Cell proliferation
Cell migation
Notch2/HEY1 pathway
14 GC scRNA-seq datasets (905186 cells and 63955 fibroblasts)
Fibroblast clustering
Fibroblast clusters functional analysis
Clinical and biological function of Cluster 6 and its key marker MFAP5

Supplementary figure 1

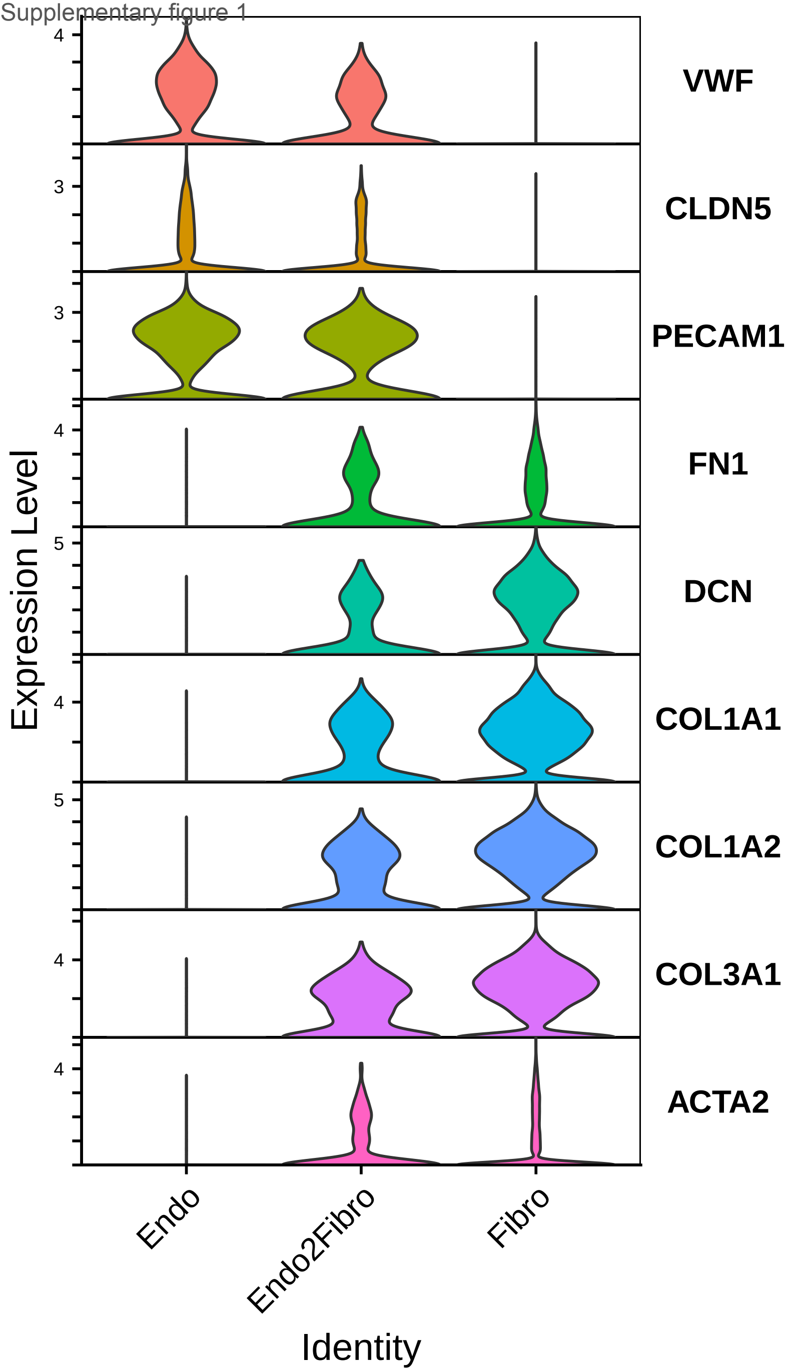

Supplementary figure 2

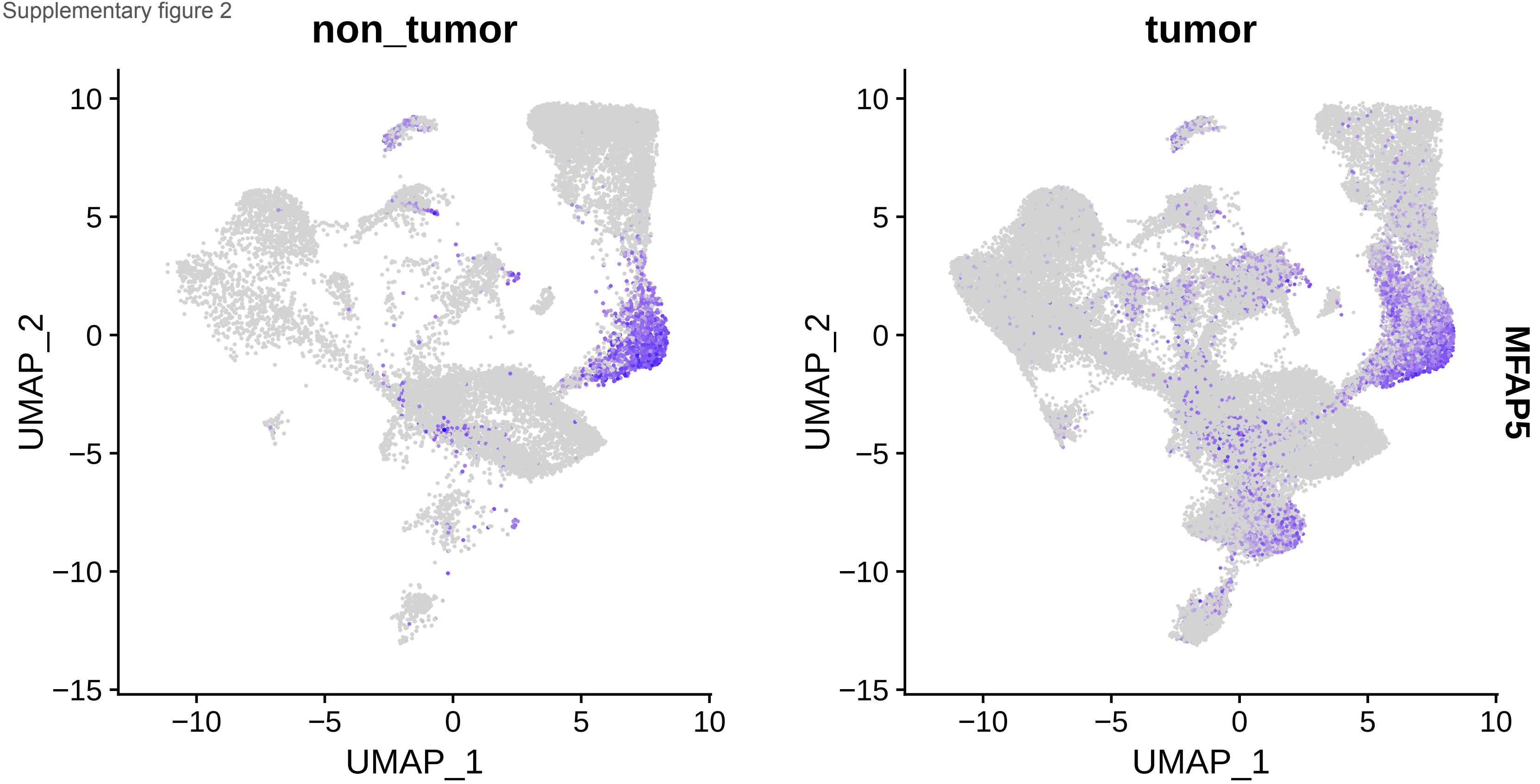

Supplement: Supplementary file 1 [file DataSheet1.pdf]
